# Rib osteochondroma with intraspinal extension and cord compression in chlidren: case report and literature review

**DOI:** 10.3389/fped.2025.1454139

**Published:** 2025-07-02

**Authors:** Weihua Ye, Guanghui Zhu, Zheng Liu

**Affiliations:** Orthopedic Department, Hunan Provincial Key Laboratory of Pediatric Orthopedics, The Affiliated Children’s Hospital of Xiangya School of Medicine, Central South University (Hunan Children’s Hospital), Changsha, China

**Keywords:** rib osteochondroma, spinal cord compression, laminectomy, vertebral fusion, internal fixation

## Abstract

**Objective:**

To report a rare case of costal osteochondroma resulting in spinal cord compression in a 5-year-old patient, and to review the existing literature on costal osteochondromas.

**Methods:**

A retrospective review was conducted on a case involving a 5-year-old male patient with hereditary multiple exostoses (HME), presenting with acute paraparesis due to a costal osteochondroma. The tumor's origin within the rib, associated myelopathic symptoms, and extensive erosion and fusion of vertebral elements were notable. The patient underwent total laminectomy, resection of the tumor, and thoracic fixation and fusion. A comprehensive literature review was performed using the keywords “Rib Osteochondroma” and “Spinal cord compression” to search the PubMed database.

**Results:**

A dumbbell-shaped bony tumor originating from the left seventh rib at the costovertebral junction was identified, causing intraspinal and extraforaminal mass effect and spinal cord compression. Surgical intervention included total laminectomy and tumor excision, followed by thoracic fixation and fusion. Histopathological analysis confirmed the diagnosis of osteochondroma. Postoperative recovery was uneventful, with significant improvement in neurological symptoms and complete resolution of lower extremity weakness at the ten-month follow-up. A mere nine cases of such presentation have been documented in the corpus of English-language literature.

**Conclusion:**

This case highlights the rarity and clinical significance of costal osteochondromas causing spinal cord compression, particularly in a young pediatric patient. Early recognition and surgical intervention are crucial for favorable outcomes. Comprehensive imaging and careful surgical planning are essential to ensure complete tumor excision and maintain spinal stability.

## Introduction

Osteochondromas represent the most prevalent benign bone tumors, manifesting either as solitary lesions or multiple lesions, the latter being indicative of HME with an autosomal dominant inheritance pattern (1, 2). While solitary lesions are more frequent, osteochondromas predominantly arise in the long bones and rarely involve the ribs. Costal osteochondromas account for only 1.5% of all osteochondromas, with instances of spinal cord compression due to a tumor originating from the rib head being exceedingly rare (3, 4).

Here, we present a rare case of costal osteochondromas resulting in spinal cord compression. Notably, this case represents the youngest documented instance of rib osteochondroma presenting as acute paraparesis. Additionally, we provide a review of the existing literature on costal osteochondromas.

## Methods

We conducted a retrospective review of a case characterized by several unusual aspects, including the tumor's origin within the rib, associated myelopathic symptoms, its occurrence in a 5-year-old patient, and the erosion and fusion of the vertebral pedicle, facet, and body due to tumor extension. The extensive tumor involvement necessitated total laminectomy of the lamina-facet complex of T7, the release of the enlarged costotransverse joint by resecting the adjacent rib, the complete excision of the tumor, followed by thoracic fixation and fusion.

A comprehensive literature review was performed using the keywords “Rib Osteochondroma” and “Spinal cord compression” to search the PubMed database for articles published up to May 2024.

## Case introduction

A 5-year-old male patient with a notable medical history of two previous surgeries for HME in the right chest and right extremities presented to our hospital, having been admitted two and one year prior for related issues. The patient exhibited a 19-day history of abnormal gait and impaired balance following a fall. The symptoms, acute in onset, were accompanied by tingling paresthesia in both legs. Six days post-symptom onset, the patient experienced a progressive decline in gait and increased difficulty in ambulation, attributable to worsening lower limb myodynamia. There was no reported history of sphincter disturbance. Upon physical examination, the patient exhibited incomplete motor loss, with muscle strength graded at 3/5 in both lower extremities. Sensory examination revealed hypesthesia in the lower limbs. Additionally, the patient showed hyperactive deep tendon reflexes in both lower extremities, with a positive Babinski sign.

**Figure 1:**
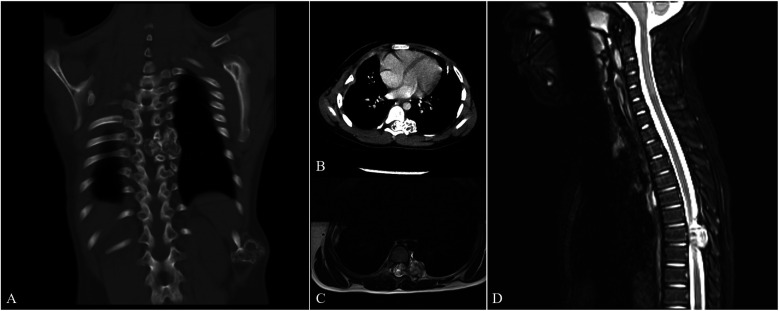
Preoperative CT and MRI images. The preoperative CT images [**(A)** coronal section, **(B)** cross section] identified a dumbbell-shaped osseous tumor emanating from the left seventh rib at the costovertebral junction, presenting as both an intraspinal and extraforaminal mass and leading to the expansion of the neural foramen at the T6/7 level. The preoperative MRI images with T2-weighted sequences [**(C)** cross section, **(D)** sagittal section] further delineated the intraspinal component, revealing a centrally mixed intermediate signal intensity and a markedly high peripheral signal intensity.

Laboratory tests returned results within normal limits. Multiplanar computed tomography (CT) revealed a dumbbell-shaped bony tumor originating from the left seventh rib at the costovertebral junction, forming an intraspinal and extraforaminal mass that expanded the neural foramen at the T6/7 level (Figures 1A,B).

Magnetic resonance imaging (MRI) with T2-weighted sequences demonstrated the intraspinal lesion with mixed intermediate signal intensity centrally and markedly high signal intensity peripherally (Figures 1C,D).

The differential diagnosis included chondrosarcoma. The presence of a thin cartilage cap (thickness < 1 cm) and the lack of any adjacent soft tissue mass formation strongly suggested a benign tumor. Nevertheless, the possibility of malignancy, such as chondrosarcoma, could not be definitively ruled out due to the patient's age and the presence of adjacent bone erosion. Consequently, *en bloc* surgery was chosen as the preferred treatment approach.

A posterior midline skin incision was utilized to expose the posterior elements from T6 to T8, with the exposure extending to encompass both ribs. The patient subsequently underwent a posterior thoracic laminectomy.

## Results

**Figure 2:**
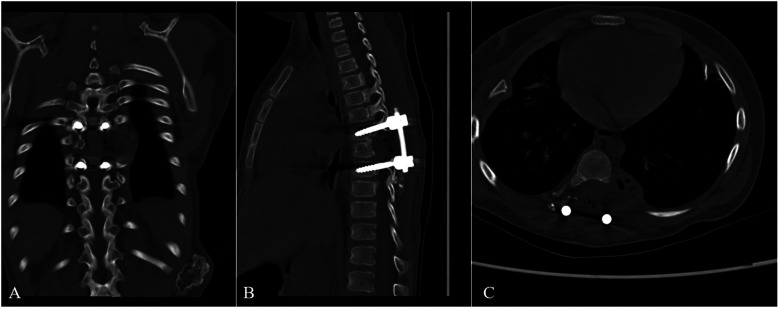
Immediately Postoperative CT images. The postoperative CT images [**(A)** coronal section, **(B)** sagittal section, **(C)** cross section] showed that the tumor had been completely removed, and the internal fixation instrument was in good position.

Operative findings revealed an atrophic spinal cord that had been displaced posterolaterally by the bony lesion originating from the left seventh rib at the costovertebral junction. During the surgical intervention, a prominent osteochondroma originating from the seventh left rib was observed intruding into the spinal canal through the intervertebral foramen, exerting a voluminous occupation of 90% of the spinal canal's dimensions. This intrusion led to a dorsal compression of the spinal cord towards the right aspect of the spinal canal, culminating in the cessation of spinal cord pulsations. The dimensions of the tumor were measured to be approximately 1.5 cm × 1 cm × 1 cm, characterized by a cauliflower-shaped surface projection with translucent cartilaginous, devoid of any discernible adherence to the adjacent tissues. Notably, the proximal extremity of the rib manifested an enlargement attributed to the presence of the osteochondroma, encompassing the left thoracic seventh intercostal space and contiguous rib. Dissection beneath the periosteum exposed the proximal rib, using an ultrasonic bone scalpel, the rib was cut along its normal course, freeing the enlarged costotransverse joint. Additionally, an atypical cauliflower-shaped tumor, approximately 2 cm × 3 cm × 3.5 cm in dimensions, was identified extrinsic to the intervertebral foramen, characterized by a sleek surface devoid of any adhesion to the surrounding tissues. Following the complete excision of the tumor, there was a progressive restoration of spinal cord volume and pulsatile activity. Subsequently, internal fixation and fusion procedures were undertaken (Figure 2).

**Figure 3:**
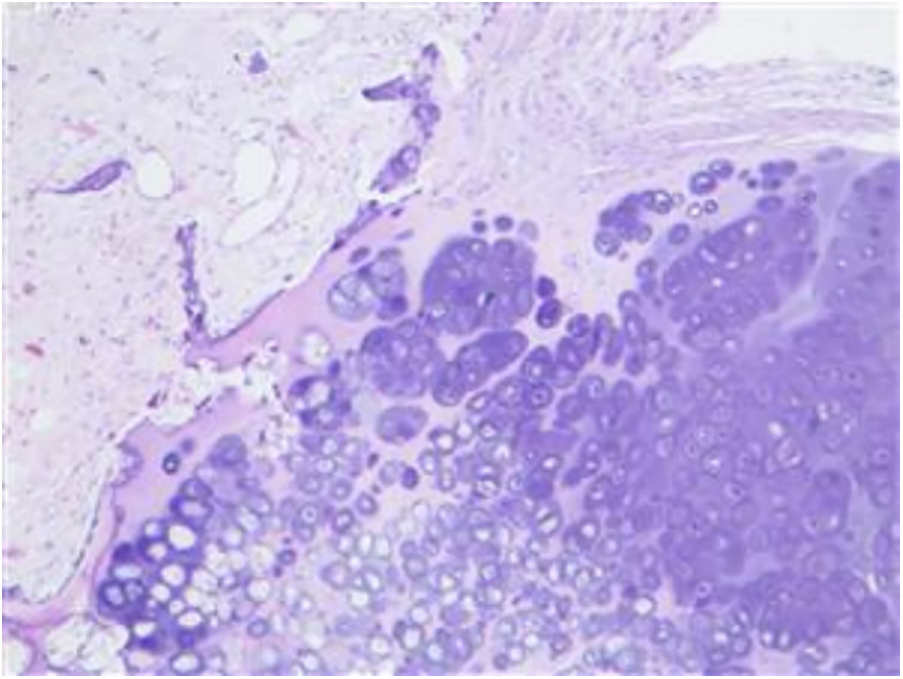
Photomicrograph of the tissue specimen. Photomicrograph of the tissue specimen demonstrates a typical osteochondroma. A cartilage cap overlying the bony trabeculae was observed, and a cellular zone of chondroosseous transformation was seen at the interface. Hematoxylin and eosin (H & E) stain; original magnification, 20×.

Histopathological analysis of the surgical specimen corroborated the diagnosis of osteochondroma (Figure 3).

The patient's postoperative convalescence proceeded without noteworthy complications. Within a week post-surgery, progressive amelioration of paraparesis and reduction in sensory deficits were observed, enabling ambulation with assistance. Discharge occurred by the seventh day post-operation. At the ten-month follow-up evaluation, complete resolution of lower-extremity weakness was documented.

A mere nine cases of such presentation have been documented in the corpus of English-language literature (Table 1) (3–10). The age of those 9 patients ranged from 12 to 58 years, most of them were younger than 30 years.The male-to-female ratio was 5:4. Eight of the patients had severe neurological deficits, and a posterior approach including laminectomy was chosen for surgical intervention. All patients showed clinical improvement during the recent follow-up period after the tumor was entirely excised.

**Table 1:** Summary of the 9 previously reported cases of costal osteochondroma causing spinal cord compression.

Cases	Gender	Age (years)	Original rib	Surgical techniques	Final follow-up (months)	Outcome	Recurrence
Twersky et al.	M	12	5th	Laminectomy	No reported	Completely recovered	No reported
Twersky et al.	F	11	4th	Laminectomy	6	Partially recovered	No reported
Natarajan et al.	M	21	5th	Thoracotomy	6	Completely recovered	No
Kane et al.	F	17	10th	Laminectomy	3	Completely recovered	No
Tang et al.	F	16	12th	Laminectomy + Facetectomy	19	Completely recovered	No
Rao et al.	F	12	6th	Laminectomy	No reported	Partially recovered	No reported
Chazono et al.	M	23	5th	Laminectomy	6	Completely recovered	No
Shim et al.	M	58	12th	Laminectomy + Facetectomy + Internal fixation + Fusion	12	Completely recovered	No
Kumar et al.	M	26	5th	No reported	12	Completely recovered	No

## Discussion

The osteochondroma variant delineated in this case report, characterized by its neural foraminal extension and resultant compression of the spinal cord, is exceptionally uncommon. During the latest postoperative monitoring phase, all subjects displayed discernible improvement in their clinical manifestations following the total excision of the neoplastic lesion. Osteochondroma manifests as a disorder of osseous growth, typically manifesting in pediatric populations (11). Tumor expansion initiates during early childhood and commonly ceases upon epiphyseal closure post-puberty (3). In the current instance, the patient, aged 5, represents the youngest documented case of costal osteochondroma.

Standard CT and MRI examinations play a pivotal role in the diagnosis and assessment of costal osteochondroma, facilitating the identification of tumor origin, dimensions, and degree of spinal canal involvement. While multiplanar CT reconstructions reliably depict the osseous component of osteochondroma, the precise tumor size may be underestimated due to the invisibility of the cartilaginous cap. Cartilage visualization is optimal on MRI, where it exhibits intermediate signal intensity on T1-weighted images and markedly heightened signal intensity on T2-weighted images. Cartilage caps exceeding 3 mm in thickness can be consistently discerned on MRI. Conversely, on T2-weighted images, the cap may appear akin to neighboring soft tissues such as epidural fat or cerebrospinal fluid, especially when the tumor encroaches upon the spinal canal. A distinctive observation in this instance is the manifestation of high signal intensity surrounding the tumor on axial T2-weighted images, potentially indicative of the cartilage cap, thereby facilitating accurate preoperative diagnosis.

Asymptomatic osteochondromas may be managed conservatively, while symptomatic manifestations necessitate surgical intervention. Typically observed in juveniles, osteochondromas primarily afflict individuals with developing skeletal systems. Their growth trajectory commences early in childhood, often ceasing post-puberty concurrent with epiphyseal closure. Surgical excision stands as the recommended therapeutic modality for symptomatic presentations. Notably, costal osteochondromas instigating neurological impairments warrant expedited excision irrespective of patient age due to their propensity for neural foraminal traversal, culminating in cord compression.

Operative procedures addressing such lesions entail meticulous consideration of potential complications. Traditional methodologies, like laminectomy, while proficient in accessing the spinal canal, suffer from inherent limitations, particularly in addressing the extraforaminal component. Supplemental facetectomy, although utilized to circumvent these deficiencies, poses risks of iatrogenic spinal instability and kyphosis over the postoperative continuum. The strategic imperative, therefore, lies in a meticulously devised surgical blueprint to ensure complete tumor eradication while averting recurrence (1).

In our study, a total laminectomy of the lamina-facet complex and the release of the enlarged costotransverse joint by resecting the adjacent rib, facilitated by the use of an ultrasonic bone scalpel, enabled extensive exposure of both intraspinal and extraforaminal components of the tumor originating from the rib.

**Figure 4:**
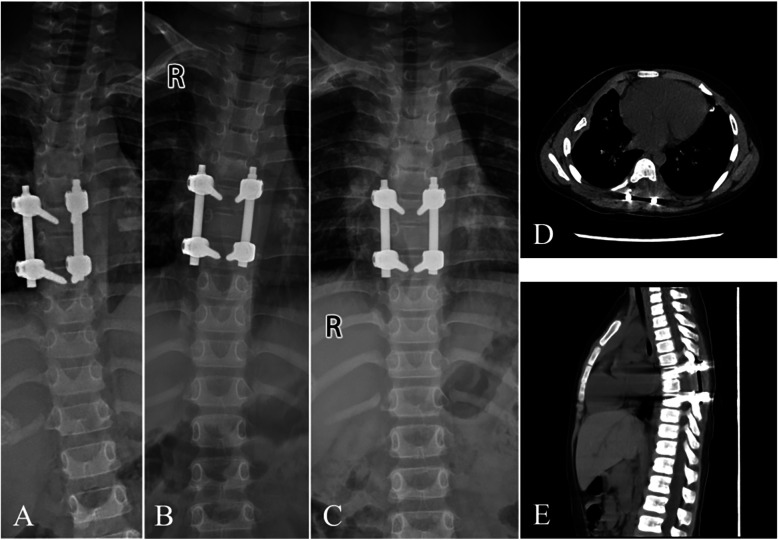
Postoperative x-ray and CT images during the follow-up. During the follow-up, at 1 week **(A)** and 1 month **(B)**, x-ray demonstrated mild scoliosis without signs of fixation failure. 5 months **(C)** after surgery, x-ray presented solid bone fusion with complete correction of scoliosis. At the last visit (10 months after surgery), CT illustrated no evidence of tumor progression with good fixation position **(D,E)**.

Consequently, posterior fixation and fusion were imperative to ensure spinal stability. Postoperative assessments using plain dynamic radiographs indicated successful bone union and correction of scoliosis at five months. Furthermore, a CT scan conducted ten months postoperatively demonstrated no evidence of tumor progression (Figure 4). Nonetheless, ongoing clinical and radiological surveillance is imperative to detect potential tumor recurrence and to identify any late-onset instability or resultant spinal deformity.

## Conclusions

This report highlights an exceptionally rare case of costal osteochondroma in a 5-year-old patient, manifesting as acute paraparesis due to spinal cord compression. The rarity of this presentation, particularly in such a young patient, underscores the need for heightened clinical awareness and prompt intervention. A review of the existing literature indicates that such presentations of costal osteochondromas with neural foraminal extension and spinal cord compression are exceedingly uncommon, with only nine similar cases reported. This case represents the youngest documented instance, contributing valuable insights to the existing body of knowledge. This case underscores the critical importance of early diagnosis, strategic surgical planning, and diligent postoperative follow-up in managing rare instances of costal osteochondroma with spinal cord compression. The insights gained from this case can inform future clinical practice and enhance outcomes for similar cases.

## Data Availability

The original contributions presented in the study are included in the article/Supplementary Material, further inquiries can be directed to the corresponding author.
